# Unraveling the role of math anxiety in students’ math performance

**DOI:** 10.3389/fpsyg.2022.979113

**Published:** 2022-11-09

**Authors:** Febe Demedts, Bert Reynvoet, Delphine Sasanguie, Fien Depaepe

**Affiliations:** ^1^Centre for Instructional Psychology and Technology, KU Leuven, Leuven, Belgium; ^2^IMEC Research Group ITEC, KU Leuven, Leuven, Belgium; ^3^Brain and Cognition, KU Leuven, Leuven, Belgium; ^4^Research Centre for Learning in Diversity, HOGENT, Ghent, Belgium

**Keywords:** math anxiety, math performance, state math anxiety, trait math anxiety, domain-specificity

## Abstract

Math anxiety (MA; i.e., feelings of anxiety experienced when being confronted with mathematics) can have negative implications on the mental health and well-being of individuals and is moderately negatively correlated with math achievement. Nevertheless, ambiguity about some aspects related to MA may prevent a fathomed understanding of this systematically observed relationship. The current study set out to bring these aspects together in a comprehensive study. Our first focus of interest was the multi-component structure of MA, whereby we investigated the relationship between state- and trait-MA and math performance (MP) and whether this relation depends on the complexity of a math task. Second, the domain-specificity of MA was considered by examining the contribution of general anxiety (GA) and MA on MP and whether MA also influences the performance in non-math tasks. In this study, 181 secondary school students aged between 16 and 18 years old were randomly presented with four tasks (varying in topic [math/non-math] and complexity [easy/difficult]). The math task was a fraction comparison task and the non-math task was a color comparison task, in which specific indicators were manipulated to develop an easy and difficult version of the tasks. For the first research question, results showed a moderate correlation between state- and trait-MA, which is independent of the complexity of the math task. Regression analyses showed that while state-MA affects MP in the easy math task, it is trait-MA that affects MP in the difficult math task. For the second research question, a high correlation was observed between GA and MA, but regression analyses showed that GA is not related to MP and MA has no predictive value for performance in non-math tasks. Taken together, this study underscores the importance of distinguishing between state and trait-MA in further research and suggests that MA is domain-specific.

## Introduction

A proper understanding of mathematics is paramount to success in one’s personal life, manifesting itself in better job opportunities and health outcomes, which also affect societal outcomes (Parsons and Bynner, 2005; OECD, 2010; Geary, 2012). This is not evident for everyone, as some people suffer from a genuine fear of mathematics (i.e., math anxiety; MA), which is especially manifested in and can affect mathematical learning during adolescence (Dowker et al., 2016; Mammarella et al., 2019). Besides the negative implications MA can have on the mental health and well-being of individuals (Mammarella et al., 2019), many empirical studies observed a consistent negative correlation between MA and math performance (MP; Namkung et al., 2019; Zhang et al., 2019; Barroso et al., 2021). Interestingly, this relation is furthermore affected by the so-called anxiety-complexity effect, which indicates a stronger link between MA and MP for more complex math tasks or domains (Punaro and Reeve, 2012; Suárez-Pellicioni et al., 2013; Huber and Artemenko, 2021).

However, there are some gaps in the current research literature on MA that impede a thorough understanding of the relationship between MA and MP. First, MA is often studied as a unidimensional construct (Lukowski et al., 2019), while MA entails a multi-component structure in which a distinction is made between MA as a state and as a trait (Wang et al., 2018; Orbach and Fritz, 2021). State-MA is a temporary and situationally based anxiety reaction, whereas trait-MA implies an acquired and relatively permanent individual predisposition. Very few existing studies differentiate between state-versus trait-MA and thereby rendering the potentially different association for these MA-components with MP has hardly been studied. Second, the domain-specificity of MA is often questioned based on the findings that MA is moderately correlated with, provokes similar symptoms as, and is associated with genetic risk factors of general anxiety (GA; Hill et al., 2016; Malanchini et al., 2017; Haase et al., 2019). People who are generally anxious may have a propensity for many forms of domain-specific anxieties, making it important to clarify the distinction between GA and MA and the relation to performance. These observations indicate that the consistently observed relationship between MA and MP can be more complex on several grounds. We want to bring these two elements together in this study and contribute to examining the factors that play a role in the association between MA and MP. The remainder of this introduction elaborates on the above-mentioned aspects and how these elements relate to MP.

### Math anxiety

Given the major importance of MA as a negative correlate of MP, MA has been intensely studied in recent years. MA can be described as the feelings of tension and nervousness some people experience when they are confronted with numerical or mathematical situations, either in educational contexts or in daily life (Ashcraft and Moore, 2009). The majority of studies measured MA in adolescents and adults using self-report questionnaires characterized by satisfactory psychometric properties (Dowker et al., 2016; Cipora et al., 2019). However, the use of various types of questionnaires and the lack of well-defined criteria to define high math-anxious individuals prevent an eloquent conclusion on the question of prevalence (Cipora et al., 2022). Based on the assumption that MA scores are normally distributed, roughly 17% of the population is highly math-anxious, which means that their MA scores are at least one standard deviation above the mean (Ashcraft et al., 2007). It was shown that as early as first grade some children already experience feelings of MA (Ganley and McGraw, 2016), but these feelings increase in students from elementary school onward (Hembree, 1990; Ramirez et al., 2013). Specific studies on the prevalence of MA in adolescents and adults suggest that the majority report mild-to-moderate levels of MA and the percentage reporting high levels of MA falls between 2 and 6% (Chinn, 2008; Hart and Ganley, 2019).

Moreover, the complexity of a math task appears to be an important factor related to MA, as more feelings of MA are experienced when completing more difficult math tasks (Anderson, 2007; Trezise and Reeve, 2018; Artemenko et al., 2021). Several indicators have been used in previous research to vary the complexity of math tasks (Artemenko et al., 2021). In particular, arithmetic is often used, with indicators of difficulty being for instance a larger number of operators, a larger number of digits, and ‘with carrying’ (as opposed to without carrying) (Hunt et al., 2017; Pizzie et al., 2020). For instance, Pizzie et al. (2020) observed that reaction times increase and accuracy decreases as the math tasks become more difficult (in this study operationalized by the indicator ‘number of operators’).

### State- and trait-math anxiety

According to the state–trait-anxiety model of Spielberger (1972), two components of anxiety can be distinguished. Applied to the concept of MA, trait-MA can be defined as a relatively stable characteristic of an individual who tends to experience feelings of anxiety when being confronted with mathematics. In contrast, state-MA refers to a temporary anxiety reaction related to a specific math situation which can fluctuate before, during, or after the specific math situation (Conlon et al., 2021). Self-reports are most commonly used to assess MA. For trait-MA, hypothetical and retrospective statements are used, whereas state-MA is often mapped through real-time questions about a current mathematical situation (Orbach et al., 2020).

Although most research focused on trait-MA, some recent studies examined the relationship between both state- and trait-MA. Results indicate that state-MA measures across different time points (i.e., offered before, during, and after a math task) are strongly related, whereas only a small-to-moderate correlation has been observed between state- and trait-MA (Orbach et al., 2019; Strohmaier et al., 2020; Conlon et al., 2021). A potential explanation for the discrepancy between state- and trait-MA, suggested by the small size of this correlation, could be that trait-MA is mainly influenced by subjective beliefs making it distinct from situation-specific state-MA experiences, as suggested by Bieg et al. (2014).

Several meta-analyses highlighted the negative relationship between MA and MP (Namkung et al., 2019; Zhang et al., 2019; Barroso et al., 2021); however, none of these meta-analyses distinguished between the effect of state-versus trait-MA and analyzed only the effects for trait-MA. Some recent studies investigated concurrently the relationship between state- and trait-MA and MP, showing contradictory findings. Conlon et al. (2021) found no significant differences between the correlation coefficients of trait-MA or state-MA measures on different time points and two MP measures (i.e., math fluency and math problem-solving task). In contrast, other studies revealed no significant correlation between trait-MA and MP (Orbach et al., 2020) or a significantly smaller correlation between trait-MA and MP than the correlation between state-MA and MP (Orbach et al., 2019).

### Domain-specificity of anxiety

By definition, MA is described as feelings of anxiety that are specifically related to mathematical situations (Ashcraft and Ridley, 2005). In contrast to this domain-specific form of anxiety, a more overarching domain-general tendency to feel anxious and to worry about everyday situations is referred to as GA (Carey et al., 2017). Both trait-MA and GA are usually measured with self-reporting questionnaires; for trait-MA, by probing the average feeling of anxiety for hypothetical mathematical situations, while for GA, the level of anxiety experienced during a certain period (usually the last 2 weeks) is gauged. The prevalence rate for GA is similar to that of trait-MA, with an estimate of 1.6 to 5% (Spitzer et al., 2006).

The domain-specificity of a specific form of anxiety, such as trait-MA, is often questioned because of several reasons. First, a consistent moderate positive is observed between trait-MA and GA (Carey et al., 2016; Field et al., 2019; Hart and Ganley, 2019; Lukowski et al., 2019). Second, both trait-MA and GA manifest in similar types of symptoms, namely affective, cognitive, physiological, and behavioral symptoms (Haase et al., 2019). Third, some studies suggest that several genetic risk factors associated with GA may be a precursor for trait-MA (Wang et al., 2014; Malanchini et al., 2017). Notwithstanding the relatedness between trait-MA and GA, previous research shows that these are two distinct constructs (Hill et al., 2016; Hart and Ganley, 2019).

In the existent evidence with regard to the question of domain-specificity of anxiety and the relation with performance, two aspects are still unclear. As trait-MA and GA are shown to be moderately correlated (e.g., Field et al., 2019), the first point concerns whether it is MA that influences MP or is it mainly overarching feelings of GA. In favor of the supposition that trait-MA has a unique predictive value for MP are studies indicating a significant correlation between trait-MA and MP when GA was partialled out (Wu et al., 2012; Hill et al., 2016). In contrast, it may be that GA plays a role in the relationship between trait-MA and MP, as shown by profile analyses demonstrating clusters of children with similar levels of GA and trait-MA (Carey et al., 2017) and studies indicating that GA affects MP (Cargnelutti et al., 2017) even when trait-MA is partialled out (Hill et al., 2016). A second question relates to whether trait-MA is solely related to MP or also to performance in other domains. Evidence on this is conflicting, while some studies found no correlation between trait-MA and for instance reading achievement (Wu et al., 2012; Hill et al., 2016; Di Lonardo Burr and LeFevre, 2021), others reported a weak negative association (Carey et al., 2017).

### Current study

Despite an increasing research interest in the domain of MA, two aspects with regard to the relation between MA and MP remain to date unclear. The first aspect concerns the multi-component structure of MA with a distinction between state- and trait-MA, and the second aspect relates to the domain-specificity of anxiety which relates to the distinction between GA and MA. Within these two issues, the complexity of math tasks or domains might play a moderating role. So far, a comprehensive study that brings all aspects together is missing. Therefore, the present study aims to gain clarity on the different factors that could potentially play a role in the consistently observed relationship between MA and MP. This study addresses this issue by measuring both state and trait MA and both domain-general GA and domain-specific MA as well as by setting up a systematic experiment with an easy and difficult version of both a math and non-math task.

Our first research question investigates the relationship between state- and trait-MA and MP and whether this relation depends on the complexity of a math task. Therefore, we developed a design that distinguishes between an easy and difficult math task. We expected state- and trait-MA to be positively correlated (Orbach et al., 2020; Strohmaier et al., 2020; Conlon et al., 2021). Further, we expected both state-MA and trait-MA to be negative predictors of students’ MP (Namkung et al., 2019; Zhang et al., 2019; Barroso et al., 2021; Conlon et al., 2021), with a better prediction of the MP in the difficult math task compared to the easy math task, relying on the assumption of the anxiety-complexity effect (Conlon et al., 2021; Huber and Artemenko, 2021).

Our second research question concerns the domain-specificity of anxiety, whereby we are interested in the contribution of GA and MA on MP and whether MA also influences the performance in non-math tasks. For this reason, in addition to the complexity dimension, we also implemented a topic dimension in our design. So, in addition to the easy and difficult math task, we also created an easy and difficult non-math task. In line with previous research, we hypothesized a positive relationship between GA and trait-MA (e.g., Hill et al., 2016; Field et al., 2019; Hart and Ganley, 2019). Further, we expected trait-MA to be a better predictor in math tasks than GA (Wu et al., 2012; Hill et al., 2016; Cargnelutti et al., 2017), while for non-math tasks we expected no predictive value from trait-MA and only from GA (Wu et al., 2012; Hill et al., 2016; Di Lonardo Burr and LeFevre, 2021).

## Methodology

### Participants

In this study, all students attending their final year of secondary education were recruited across two secondary schools in Flanders. Both schools provide general secondary education but offer both mathematical and non-mathematical tracks, resulting in a variety in the sample in terms of students’ mathematical competencies. Regardless of differences in the mathematical abilities of the participating students, all students had the necessary cognitive skills to deal with the assignments. A total number of 185 students took part in this study, but four students had to be excluded from the sample due to incomplete measurements. The resulting sample included 181 students (87 males) aged between 16 and 18 years old, with an average age of 16.87 (*SD* = 0.43). Based on pilot data, *a priori* sample size calculation *via* Gpower-3.1.9.4 software was conducted, recommending a sample size of 156 to yield a power of 0.95.

### Procedure

Informed consent was signed before participation and data were collected according to the regulations of and approved by the KU Leuven’s Social and Societal Ethics Committee (G^−2021^-3,868). Students were tested in the classroom in group sessions lasting approximately one school hour. First, four self-rating questionnaires were administered in a fixed order to assess some trait constructs: general self-concept, math self-concept, GA, and trait-MA. Then, four tasks varying by complexity (easy/difficult) and topic (math/non-math) were administered in a randomized order. After each task, levels of state anxiety and state self-concept were rated. Both trait and state self-concept measures were not further analyzed because they are beyond the scope of this study.

### Computerized tasks

All students were presented with four computerized comparison tasks in a randomized order: (1) easy math task, (2) difficult math task, (3) easy non-math task, and (4) difficult non-math task. The math task was a fraction comparison task where students had to answer which fraction was the largest. To create an easy and difficult task, several characteristics of the fractions were manipulated that have been shown to affect the complexity of fraction comparisons, more specifically natural number bias, distance effect, gap thinking, benchmarking, common components, and number of digits (DeWolf and Vosniadou, 2011; Faulkenberry and Pierce, 2011; Vamvakoussi et al., 2012; Gómez et al., 2015). In the supplemental materials, we elaborate on each of these indicators and illustrate a concrete example (see Supplementary Figure 1). For the non-math task, a color comparison task was developed. The instruction in the non-math task was to indicate on which side of the screen the mixing of the colors resulted in the darkest color (see Supplementary Figure 2). Similar to the math task, four elements must be processed and combined in a forced comparison decision. Despite the similarity in task design, the tasks are different because the math task demands semantic reasoning and the non-math task relies on perceptual reasoning.

The tasks were offered through the program Psychopy. Each trial consisted of two stimuli presented on the opposite sides of the screen. Each trial was an image with a size of 30×25 pixels. Participants were instructed to judge which stimulus was larger/darker (for math and non-math tasks, respectively) by pressing the right and left arrow keys. After a response was given, a fixation cross was displayed at the center of the screen to separate trials with an interstimulus interval of 1,400 ms. Five practice trials were provided with feedback on accuracy. The practice trials were followed by 35 randomly presented experimental trials. Accuracy and response time was logged for each trial. It is important to notice that within this paper, MP were operationalized as the average accuracy and response time across the 35 items of a task. All tasks are available.^1^

### Measurement instruments

#### Trait measures

To measure the trait constructs, questionnaires were administered online through Qualtrics. All questionnaires were translated to Dutch and had to be answered using a Likert scale, with higher scores indicating higher levels of the surveyed construct.

General Anxiety. GA was measured with the Generalized Anxiety Disorder Questionnaire (GAD-7; Spitzer et al., 2006), which exists of seven items (e.g., Feeling nervous, anxious, or on edge) requiring participants to rate how frequently they experience this feeling of GA, going from not at all (1) to nearly every day (4). The reliability for this scale was α = 0.88.

Trait Math Anxiety. Trait-MA was measured with the Abbreviated Math Anxiety Scale (AMAS; Hopko et al., 2003). Participants had to rate how anxious they usually feel in nine mathematical contexts (e.g., Taking an examination in a math course), ranging from low anxious (1) to high anxious (5). The reliability for this scale was α = 0.88.

#### State measures

State measures were probed retrospectively after each task on a scale from one to then. State anxiety was assessed by the question “How anxious were you while doing the exercises?.” Scoring ranged from not anxious (1) to very anxious (10).

### Statistical analyses

The following sections discuss in detail the Pearson’s correlation analyses and linear multiple regression analyses that were conducted to address the two research questions. All statistical analyses were conducted using SPSS Statistics 28. In line with Cohen (1988), we consider correlation values of *r* ≥ 0.1 small, *r* ≥ 0.3 medium, and *r* ≥ 0.5 large. For the linear multiple regression analyses, the enter method was used, a procedure whereby the selected independent variables are entered in one step.

The first research question investigates the relationship between state- and trait-MA and analyzes how they uniquely contribute to MP. Therefore, Pearson’s correlation analyses were conducted between state- and trait-MA and MP for both the easy and difficult math task. Moreover, four multiple linear regression analyses were conducted with MP (i.e., accuracies and reaction times for the easy and difficult math tasks) as dependent variables and state- and trait-MA as possible predictors.

The second research question was to investigate the relationship between domain-general GA and domain-specific MA and explore how they uniquely contribute to performance. Pearson’s correlation analyses were used to explore the relationships between GA, trait-MA, and performance in math tasks (i.e., MP) and non-math tasks. Related to the issue of domain-specificity, we aimed to answer two subquestions. First, to examine if the variance in MP can be explained by domain-specific anxiety (i.e., trait-MA) or also by an overarching anxiety (i.e., GA), four linear multiple regression analyses were conducted with MP as dependent variables (i.e., accuracies and reaction times for the easy and difficult math tasks) and trait-MA and GA as possible predictors. Second, to investigate if trait-MA is solely related to MP, four linear multiple regression analyses were conducted with performance in the non-math tasks as dependent variables (i.e., accuracies and reaction times for the easy and difficult non-math tasks) and trait-MA and GA as possible predictors.

## Results

The values for GA ranged from 7 to 28 with an average of 13.81 (*SD* = 4.92) and for trait-MA from 9 to 42 with an average of 21.60 (*SD* = 6.32). Descriptive statistics (means and standard deviation) for raw values on the accuracies, reaction times, and state anxiety for each task are reported in Table 1. Bivariate correlations among the variables in the math tasks are shown in Table 2.

**Table 1:** Descriptive Statistics [Mean (Standard Deviation)] for Accuracies (with a maximum of 35), Reaction Times and State Anxiety (with a maximum of 10).

	Easy	Difficult	Easy	Difficult	Easy	Difficult
	Accuracies		Reaction times		State anxiety	
Math content	33.51 (2.38)	24.03 (4.53)	1.67 (0.97)	5.51 (4.00)	2.85 (2.20)	3.79 (2.48)
Non-math content	34.90 (0.37)	23.96 (4.48)	0.62 (0.25)	2.29 (1.14)	2.15 (2.09)	3.08 (2.32)

**Table 2:** Correlation matrix for math tasks.

	1	2	3	4	5	6	7	8
Accuracy easy math								
Accuracy difficult math	0.35^***^							
Reaction time easy math	−0.05	−0.33^***^						
Reaction time difficult math	0.24^**^	0.32^***^	0.19^**^					
State-MA easy math	−0.29^***^	−0.15^*^	0.09	−0.04				
State-MA difficult math	−0.15^*^	−0.14	0.03	0.01	0.69^***^			
Trait-MA	−0.17^*^	−0.20^**^	0.05	−0.10	0.45^***^	0.45^***^		
Trait-GA	−0.10	−0.06	0.04	−0.00	0.48^***^	0.39^***^	0.60^***^	

*** *p* < 0.001;

** *p* < 0.01;

* *p* < 0.05.

Before turning to the research questions, a paired samples *t*-test was conducted on the accuracies of the four different tasks to check whether the accuracies were significantly lower in the difficult tasks than in the easy tasks, as intended with the task complexity manipulation. Results showed that this was indeed the case, both for the math tasks (*t*(180) = 29.46, *p* < 0.001) and the non-math tasks (*t*(180) = 33.12, *p* < 0.001).

### State- and trait-math anxiety

A positive medium correlation was observed between state-MA and trait-MA, both in the easy and the difficult math task (i.e., both *r* = 0.45, p < 0.001). Moreover, state-MA in the easy math task and state-MA in the difficult math task were highly correlated (*r* = 0.69, *p* < 0.001). Regarding the accuracy on the easy math task, a small negative correlation was found with trait-MA (*r* = −0.17, *p* = 0.02) and a small-to-moderate negative correlation with state-MA (*r* = −0.29, *p* < 0.001). Similar results were observed for the correlation between the accuracy in the difficult math task and trait-MA (*r* = −0.20, *p* = 0.01); in contrast, we observed a small negative non-significant correlation between the accuracy in the difficult math task and state-MA (*r* = −0.14, *p* = 0.07). No significant correlations were found concerning the relation with reaction times.

The linear multiple regression models with accuracy as an outcome variable and state- and trait-MA as potential predictors were significant, both in the easy (*F*(2,178) = 8.38, *p* < 0.001) and difficult (*F*(2,178) = 3.89, *p* = 0.02) math task. In the easy math task, the model accounted for 8.6% of the variance in accuracy scores and only state-MA was a significant predictor variable (*β* = −0.27, *p* = 0.001). For the difficult math task, the model explained 4.2% of the variance in accuracy scores. In this model, state-MA was not a significant predictor variable, but trait-MA was predictive (*β* = −0.17, *p* = 0.04). For reaction time, the regression models were not significant. More information about the linear multiple regression models are included in the Appendix (see Tables 1–4).

### Domain-specificity of anxiety

A high positive correlation was observed between GA and trait-MA (*r* = 0.60, *p* < 0.001). No significant correlation was found between GA and any performance measure for math and non-math tasks. Trait-MA was significantly negatively correlated with accuracies in the math tasks, both for the easy (*r* = −0.17, *p* = 0.02) and difficult (*r* = −0.20, *p* = 0.01) task; this was not the case for the non-math tasks (i.e., easy non-math task *r* = 0.14, *p* = 0.06 and difficult non-math task *r* = 0.01, *p* = 0.85).

Related to the first subquestion, whether the variance in MP can be explained only by trait-MA or also by an overarching GA, four linear multiple regression analyses were conducted with MP as dependent variables (i.e., accuracies and reaction times for the easy and difficult math tasks) and trait-MA and GA as possible predictors. Only the regression analysis for the accuracy in the difficult math task was significant (*F*(2, 178) = 4.09, *p* = 0.02) and the predictor variables explained 4% of the variance in accuracy in the difficult math task. The only significant independent predictor was trait-MA (*β* = −0.25, *p* = 0.01). GA had no predictive value in the MP. The second subquestion was if the variance in non-math performance (i.e., accuracies and reaction times for the easy and difficult non-math tasks) can be explained by an overarching GA or if also trait-MA can explain part of the variance. The four linear multiple regressions were non-significant, meaning that neither GA nor trait-MA significantly predict the variance in non-math performance. More information about the linear multiple regression models are included in the Appendix (see Tables 5–12).

## Discussion

Proficiency in mathematics has far-reaching consequences for individuals’ lives and even society. Since a consistent association has been observed between MA and MP (e.g., Barroso et al., 2021), the construct of MA has gained increasing research attention in latest years. Although some recent studies have provided renewed insights regarding MA, there is still uncertainty about several aspects related to the association between MA and MP. Therefore, this study aimed to gain clarity on different factors that could potentially play a role in the consistently observed relationship between MA and MP. First, we examined the relationship between state- and trait-MA and MP and whether this relation depends on the complexity of a math task, relying on the assumption of the anxiety-complexity effect. Second, we investigated the domain-specificity of anxiety in the relation between MA and MP by examining the relative contribution of trait-MA versus GA to MP on the one hand, and on the other hand by examining whether trait-MA only contributes to the performance in math tasks, or also influences the performance in non-math tasks.

### State- and trait-math anxiety

In line with previous research (Strohmaier et al., 2020; Conlon et al., 2021), results from the current study showed a moderate correlation between state- and trait-MA, which remained constant for the easy and difficult math tasks. This indicates that students with high trait-MA also reported high state-MA. A plausible explanation for this result might be that the experiment was conducted in a low-stakes environment. This resulted in rather low scores for state-MA (e.g., 2.85 for the easy math task and 3.79 for the difficult math task), preventing large differences between state-MA scores for easy and difficult math tasks. Despite the moderate correlation between state- and trait-MA, we found a high correlation between the two state-MA measurements. This replicated result (Conlon et al., 2021) suggests that state- and trait-MA are two distinct components of MA.

Next, we sought to examine the relationship between state- and trait-MA and MP. The reaction times were not related to any MA measure. This study supports evidence from previous meta-analyses (Namkung et al., 2019; Zhang et al., 2020; Barroso et al., 2021) by showing a comparable negative correlation between trait-MA and MP (i.e., accuracies). State-MA was only significantly related to accuracies in the easy math task and not significantly related to accuracies in the difficult math task. Surprisingly, the multiple linear regression analyses of MP with state- and trait-MA being possible predictors indicated that state-MA was a significant predictor for the accuracies in the easy math task, whereas trait-MA was a significant predictor for the accuracies in the difficult math task. Contrary to our expectation based on the anxiety-complexity hypothesis, results showed that there was twice as much explained variance in the model of accuracies in the easy math task compared to the difficult math task. These results are extremely interesting, as they suggest that MA explains a significant part of the variance in MP, even regarding easy math tasks.

These results are partly in line with the studies by Orbach et al. (2019, 2020) showing that only state-MA is related to MP and is explaining the variance in MP, because in those studies also, a basic number skill test (i.e., easy task) was utilized to measure MP. Moreover, it should be noted that Orbach and colleagues (2019; 2020) only reported a low correlation between their state- and trait-MA measures, which might explain why no significant correlation was found with trait-MA. In the present study, MP was measured in both an easy and difficult math task, revealing different relationships with MA measures. A possible explanation for the finding that state-MA only plays a role in the relation to MP in the easy math task can be found in the definition, since state-MA is a temporary anxiety response related to a specific situation. In contrast, trait-MA is a more permanent personality trait related to mathematics and is indeed related to MP in both math tasks. However, further research is needed to explore these different associations between the MA-components with MP.

### Domain-specificity of anxiety

Our second research question relates to the domain-specificity of MA. In line with previous research, we found a positive correlation between GA and trait-MA (e.g., Field et al., 2019). However, the observed association in this study was high (*r* = 0.60), whereas previous research reported only moderate correlations (e.g., Field et al., 2019 (*r* = 0.35); Malanchini et al., 2017 (*r* = 0.32)). Although the same measurement instruments were used, the results may be influenced by the translation of the questionnaire. Moreover, the sample in this study is slightly younger (i.e., aged between 16–18 year) compared to the studies by Field et al. (2019) and Malanchini et al., 2017 (i.e., aged between 18 and 21 years), and furthermore, it is feasible that cultural influences have an impact in this relationship (Dowker et al., 2016).

Regarding the first subquestion about the relationship between trait-MA, GA, and MP, we observed no significant correlations between GA and MP, whereas a negative, although small correlation, was observed between trait-MA and accuracies in both math tasks. Furthermore, the accuracies in the difficult math task were significantly predicted by trait-MA, but not by GA. For the accuracies in the easy math task, we know based on the previous research question, it was not trait-MA that predicted the accuracies, but rather state-MA. The reaction times were not related to trait-MA, nor GA. These findings indicate that a domain-specific, rather than a general form of anxiety predicts performance in the domain of mathematics.

Another subquestion relates to whether MA has an impact only on MP or also on performance in other domains. In this study, no correlations were observed between trait-MA and GA, on the one hand, and performance in the non-math tasks, on the other hand. Furthermore, trait-MA and GA did not significantly predict the accuracies in the non-math tasks, none of the entered predictors in the model were significant. These findings confirm earlier results by showing no association between trait-MA and performance in other domains, such as for instance reading achievement (e.g., Wu et al., 2012; Hill et al., 2016; Di Lonardo Burr and LeFevre, 2021). An exception is the study of Carey et al. (2017) reporting a weak negative association between trait-MA and reading achievement. This absent relationship between trait-MA and performance in non-math tasks can be interpreted as supporting evidence for the domain-specificity of MA.

### Limitations

Several limitations of this study should be noted, although we attempted to minimize their impact. First, this is an experimental study that prevented the use of ecologically valid mathematical situations. Consequently, state-MA responses probably did not appear as strong as they do in ecologically valid mathematical settings. However, to account for ecological validity, the experiment was conducted in authentic classrooms in the attendance of the teacher instead of in a laboratory context impeding a possible bias of ecological validity. A second limitation concerns the applied self-report measurements, since the criticism on this type of measurement is that there is likely to be some bias due to the complexity of consciously making these types of judgments and socially desirable response behavior (Dowker et al., 2016). We attempted to minimize this potential bias by offering the self-reported state questionnaires immediately after the task was completed and assuring students through informed consent that their responses will be treated confidentially. In future research, we will explore the potential of alternative measurement methods (e.g., physiological responses). A third limitation is the correlational nature of this study design, preventing the ability to draw causal inferences. Subsequently, these limitations may prompt, in follow-up research, the use of different measurement methods to capture state-MA or to implement this research focus in a longitudinal research design. A fourth limitation relates to the developed 2-by-2 design for which we varied the difficulty (easy vs. difficult) of a math and non-math task. It might be argued that in addition to the subject matter, these tasks also differ in terms of the degree of required reasoning, since the math task is semantic in nature, whereas the non-math task is rather perceptual. However, to take this into account, instructions, format, and response options were kept as similar as possible in both tasks. Further research can account for this difference, for instance, by adding another control task that similarly to the math task requires semantic reasoning (e.g., language task).

## Conclusion

Although our results replicate the consistently observed association between MA and MP, the present study extends previous research on the relation between MA and MP. First, our findings indicate that the relationship between state- and trait-MA is independent of the complexity of the math task. Moreover, this study shows that despite the moderate correlation between state- and trait-MA, both constructs are distinguishable. Results indicate that state-MA affects MP in easy math tasks and trait-MA affects MP in more difficult math tasks. Second, while MA and GA are highly correlated, it was observed that GA is unrelated to MP and that performance in a non-math task is not affected by MA. Together these results highlight the importance of distinguishing between state- and trait-MA in further research and suggest that MA is domain-specific related to math content.

## Data availability statement

The data that support the findings of this study are available from the corresponding author, upon reasonable request.

## Ethics statement

All procedures were performed in accordance with the ethical standards of the institutional research committee and approved by the Social and Societal Ethics Committee (SMEC) of the KU Leuven (G-2021-3868). Written informed consent was obtained from all participants for their participation in this study.

## Author contributions

FD was responsible for the data collection, statistical analysis, the manuscript and the editing. All authors contributed to conception and design of the study, manuscript revision, read, and approved the submitted version.

## Funding

This research was funded by the Research Foundation—Flanders under Grant G068520N.

## Conflict of interest

The authors declare that the research was conducted in the absence of any commercial or financial relationships that could be construed as a potential conflict of interest.

## Publisher’s note

All claims expressed in this article are solely those of the authors and do not necessarily represent those of their affiliated organizations, or those of the publisher, the editors and the reviewers. Any product that may be evaluated in this article, or claim that may be made by its manufacturer, is not guaranteed or endorsed by the publisher.

## Footnotes

^1^ https://osf.io/pesz7/?view_only=008bedbc56bb4e89b3e5326d8e7fa207
