# Supplementary material for: Unraveling the role of math anxiety in students’ math performance
Source: Front Psychol. 2022 Nov 9;13:979113. doi: 10.3389/fpsyg.2022.979113 (PMC9682958; doi:10.3389/fpsyg.2022.979113)
Supplement: Supplementary file 1 [file Presentation_1.pdf]

Supplementary Material

Figure 1

Manipulation of the Fraction Comparison Task (Math Task)

EASY MATH CONDITION			DIFFICULT MATH CONDITION		
$\frac{2}{7}$ $\frac{2}{3}$	Natural number bias	Incongruent	$\frac{13}{19}$ $\frac{17}{26}$	Natural number bias	Incongruent
	Distance effect	Large		Distance effect	Small
	Gap Thinking	Correct		Gap Thinking	Correct
	Bench marking	Yes		Bench marking	No
	Components	Common components		Components	Without common components
	Digits	Single digits		Digits	Double digits

Easy

The easy trial will be solved correctly if the gap thinking or benchmarking strategy is used. This trial is easy because it is single digit, consists of common components and there is a large distance effect. When relying on the natural number bias this item will be solved incorrectly.

Difficult

This trial will be solved incorrectly if the natural number bias or benchmarking strategy is used. Only when relying on the gap thinking strategy, this item will be solved correctly. This trial is difficult because it consists of double digits, without common components and the distance effect is small.

Mathematical indicators

(1) Natural number bias
A major cause of students' difficulties with fractions is due to natural number bias, meaning that students tend to rely on natural number principles when reasoning about fractions, even when this is not justified (Ni & Zhou, 2005; Obersteiner et al., 2013; Reinhold et al., 2020; Vamvakoussi et al., 2012). Accordingly, students perform better on items where the largest fraction also has the largest numerator and denominator (i.e. congruent items, for example, 2/4 vs 1/3). Moreover, evidence for the natural number bias was found in that correct answers to incongruent items (i.e. where the natural number bias leads to the incorrect solution) took longer than giving a correct answer to congruent items (i.e. where the natural number bias leads to the correct solution) (Vamvakoussi et al., 2012; Van Hoof et al., 2013).

(2) Distance effect

It was shown that the mean reaction time for fraction comparison tasks was predicted by the numerical distance between fractions. In other words, as two fractions are further apart, the median time to respond on the fraction comparison task decreases (Faulkenberry & Pierce, 2011). In this sense, fractions whose magnitude are closer together may be perceived as more difficult than fractions that are further apart. We distinguished between a small (≤ 0.15), medium (>0.15 - <0.35), and large distance (≥ 0.35).

(3) Gap thinking

When responding to a fraction comparison task, many individuals apply the so-called gap thinking strategy, which consists of relying on which fraction has the smallest difference between the numerator and denominator to determine which fraction is the largest (González-Forte et al., 2020). For example, when comparing 2/7 versus 2/3, the gap between 2 and 7 is 5 which is more than the gap of 1 between 2 and 3, from which, according to this strategy, one concludes that 2/3 is larger than 2/7. For this indicator, it is considered whether applying the gap thinking strategy results in a correct answer or not. An additional neutral category was added because some items have the same gap (e.g. 6/7 vs 1/2).

(4) Benchmarking

Some fractions, such as 1/2, are very prevalent so they can act as so-called benchmarks because their numerical value is instantly known (Obersteiner et al., 2020). When one of the two to be compared fractions in a fraction comparison task is a benchmark, the task proved to be easier. For a fraction comparison task, benchmarks may also be helpful when the two fractions to be compared are located on either side of a benchmark, for example, 7/9 is larger dan 1/2 and 3/8 is smaller than 1/2. We selected 9 benchmark fractions, so when one of the fractions of the comparison task was one of the 9 benchmarks, this indicator was categorized as easy. Furthermore, we made a distinction if the task contains no benchmark, but the two fractures to be compared were on either side of 1/2, the category was coded as medium and otherwise as difficult.

(5) Components

Research on fraction comparison tasks has shown that fractions with a common component lead to more correct answers than fractions without a common component (González-Forte et al., 2020). In addition, it has been found that a common denominator (e.g. 1/4 vs 3/4), in particular, results in more correct fracture equations compared to common numerators (e.g. 2/4 vs 2/6). This indicator was coded as easy for fractions with a common component and difficult for fractions without a common component.

(6) Digits

It is hypothesized that fractions consisting of double-digit numbers are more difficult than fractions consisting of single-digit numbers. A study focused on the natural number bias showed that for congruent fraction comparisons, 60% of the errors occurred when the fractions contained double-digit numbers (DeWolf & Vosniadou, 2011). Therefore, fractions with only single-digit numbers were coded as easy, fractions with only double-digits were coded as difficult and fractions containing a mix of single- and double digits were coded as medium.

Figure 2

Manipulation of the Colour Comparison Task (Non-Math Task)

**EASY NON-MATH CONDITION**			**DIFFICULT NON-MATH CONDITION**		
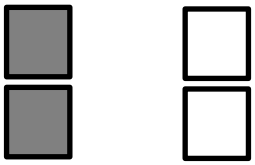	Distance effect total	Large	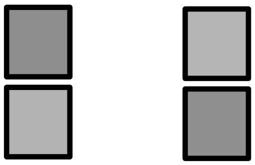	Distance effect total	Small
	Exactness brightness	Minimum differences of 10		Exactness brightness	Difference of 1
	Bench marking	Yes		Bench marking	No
	lightest/darkest	Congruent		lightest/darkest	Incongruent
	Components	Without common components		Components	Without common components
	Need to mix	No		Need to mix	Yes

Easy

This item is easy because there is a large distance effect, large brightness differences, congruency and there is no need to mix. There are no common components in this trial, but in this case it does not affect the difficulty of this trial.

Difficult

This is a difficult item because of the small distance effect, small differences in brightness of the colours, no possible benchmarks, no congruency between lightest and darkest colour, no common components and the need to mix.

Figure 3

Phased Progress of the Trials (Example for the Easy Math Task)

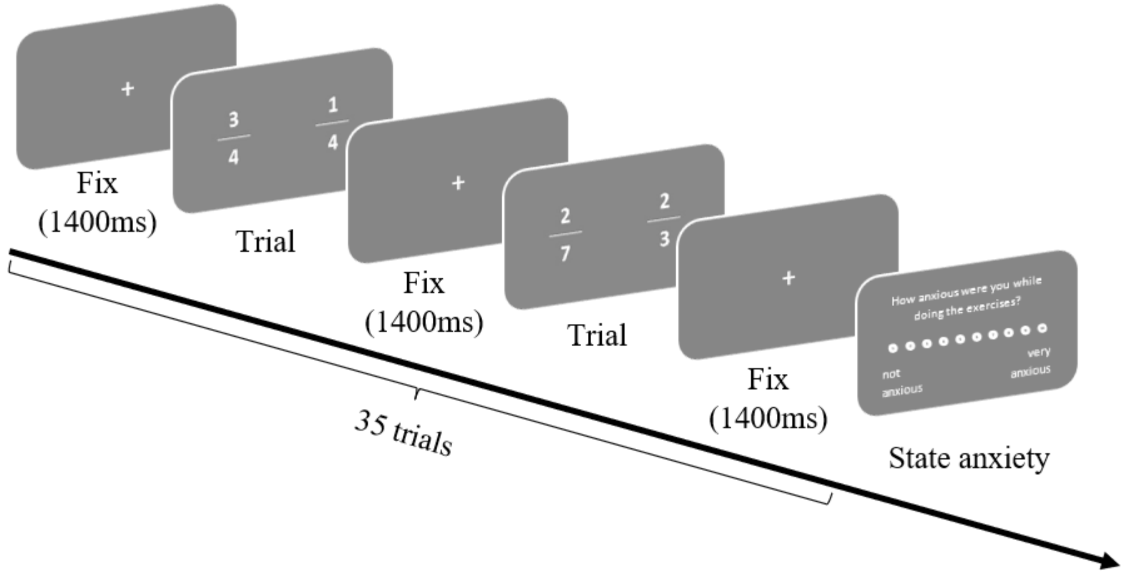

Table 1
Linear multiple regression analysis with accuracies for the easy math task as dependent variable and trait- and state-MA as possible predictors

	B	95% CI for B		SE B	β
		LL	UL		
Constant	34.75	33.54	35.95	0.61	
Trait-MA	-0.02	-0.08	0.04	0.03	-.05
State-MA	-0.29	-0.46	-0.12	0.09	-.27***

Note: $F(2,178) = 8.38$, $p < .001$, $R^2 = .09$, $R^2_{adj} = .08$
*** <.001 ; ** <.01 ; * <.05

Table 2
Linear multiple regression analysis with accuracies for the difficult math task as dependent variable and trait- and state-MA as possible predictors

	B	95% CI for B		SE B	β
		LL	UL		
Constant	27.09	24.76	29.43	1.18	
Trait-MA	-0.12	-0.24	-0.01	0.06	-.17*
State-MA	-0.11	-0.10	0.19	0.15	-.06

Note: $F(2,178) = 3.89$, $p = .02$, $R^2 = .04$, $R^2_{adj} = .03$
*** <.001 ; ** <.01 ; * <.05

Table 3
Linear multiple regression analysis with reaction times for the easy math task as dependent variable and trait- and state-MA as possible predictors

	B	95% CI for B		SE B	β
		LL	UL		
Constant	1.52	1.01	2.03	0.26	
Trait-MA	0.00	-0.02	0.03	0.01	.02
State-MA	0.04	-0.04	0.11	0.04	.08

Note: $F(2,178) = 0.68$, $p = .51$, $R^2 = .01$, $R^2_{adj} = .00$
*** <.001 ; ** <.01 ; * <.05

Table 4
Linear multiple regression analysis with reaction times for the difficult math task as dependent variable and trait- and state-MA as possible predictors

	B	95% CI for B		SE B	β
		LL	UL		
Constant	6.84	4.74	8.94	1.06	
Trait-MA	-0.08	-0.18	0.03	0.05	-.13
State-MA	0.10	-0.16	0.37	0.13	.06

Note: $F(2,178) = 1.14$, $p = .32$, $R^2 = .01$, $R^2_{adj} = .00$
*** <.001 ; ** <.01 ; * <.05

Table 5
Linear multiple regression analysis with accuracies for the easy math task as dependent variable and trait- MA and GA as possible predictors

	B	95% CI for B		SE B	β
		LL	UL		
Constant	34.91	33.63	36.20	0.65	
GA	-0.00	-0.09	0.09	0.05	-.00
Trait-MA	-0.06	-0.13	0.00	0.04	-.17

Note: $F(2,178) = 2.71$, $p = .07$, $R^2 = .03$, $R^2_{adj} = .02$
*** <.001 ; ** <.01 ; * <.05

Table 6
Linear multiple regression analysis with accuracies for the difficult math task as dependent variable and trait-MA and GA as possible predictors

	B	95% CI for B		SE B	β
		LL	UL		
Constant	26.79	24.38	29.21	1.22	
GA	0.08	-0.09	0.25	0.08	.09
Trait-MA	-0.18	-0.31	-0.05	0.07	-.25**

Note: $F(2,178) = 4.09$, $p = .02$, $R^2 = .04$, $R^2_{adj} = .03$
*** <.001 ; ** <.01 ; * <.05

Table 7
Linear multiple regression analysis with reaction times for the easy math task as dependent variable and trait-MA and GA as possible predictors

	B	95% CI for B		SE B	β
		LL	UL		
Constant	1.49	0.96	2.02	0.27	
GA	0.00	-0.03	0.04	0.02	.02
Trait-MA	0.01	-0.02	0.04	0.01	.04

Note: $F(2,178) = 0.26$, $p = .77$, $R^2 = .00$, $R^2_{adj} = .00$
*** <.001 ; ** <.01 ; * <.05

Table 8
Linear multiple regression analysis with reaction times for the difficult math task as dependent variable and trait-MA and GA as possible predictors

	B	95% CI for B		SE B	β
		LL	UL		
Constant	6.58	4.41	8.76	1.10	
GA	0.07	-0.08	0.22	0.08	.08
Trait-MA	-0.09	-0.21	0.02	0.06	-.15

Note: $F(2,178) = 1.25$, $p = .29$, $R^2 = .01$, $R^2_{adj} = .00$
*** <.001 ; ** <.01 ; * <.05

Table 9

Linear multiple regression analysis with accuracies for the easy non-math task as dependent variable and trait- MA and GA as possible predictors

	B	95% CI for B		SE B	β
		LL	UL		
Constant	34.73	34.53	34.93	0.10	
GA	-0.00	-0.02	0.01	0.01	-.06
Trait-MA	0.01	0.00	0.02	0.01	.17

Note: $F(2,178) = 1.98$, $p = .14$, $R^2 = .02$, $R^2_{adj} = .01$
*** <.001 ; ** <.01 ; * <.05

Table 10

Linear multiple regression analysis with accuracies for the difficult non-math task as dependent variable and trait-MA and GA as possible predictors

	B	95% CI for B		SE B	β
		LL	UL		
Constant	23.49	21.05	25.94	1.24	
GA	0.06	-0.10	0.23	0.09	0.07
Trait-MA	-0.02	-0.15	0.11	0.07	-0.03

Note: $F(2,178) = 0.30$, $p = .74$, $R^2 = .00$, $R^2_{adj} = .01$
*** <.001 ; ** <.01 ; * <.05

Table 11

Linear multiple regression analysis with reaction times for the easy non-math task as dependent variable and trait-MA and GA as possible predictors

	B	95% CI for B		SE B	β
		LL	UL		
Constant	0.55	0.41	0.68	0.07	
GA	0.00	-0.01	0.01	0.01	-.01
Trait-MA	0.00	-0.00	0.01	0.00	.09

Note: $F(2,178) = 0.67$, $p = .51$, $R^2 = .01$, $R^2_{adj} = .00$
*** <.001 ; ** <.01 ; * <.05

Table 12

Linear multiple regression analysis with reaction times for the difficult non-math task as dependent variable and trait-MA and GA as possible predictors

	B	95% CI for B		SE B	β
		LL	UL		
Constant	2.57	1.95	3.19	0.31	
GA	-0.01	-0.05	0.03	0.02	-.04
Trait-MA	-0.01	-0.04	0.03	0.02	-.04

Note: $F(2,178) = 0.45$, $p = .64$, $R^2 = .01$, $R^2_{adj} = .01$
*** <.001 ; ** <.01 ; * <.05
